# Cryogenian Glacial Habitats as a Plant Terrestrialisation Cradle – The Origin of the Anydrophytes and Zygnematophyceae Split

**DOI:** 10.3389/fpls.2021.735020

**Published:** 2022-01-27

**Authors:** Jakub Žárský, Vojtěch Žárský, Martin Hanáček, Viktor Žárský

**Affiliations:** ^1^CryoEco Research Group, Department of Ecology, Faculty of Science, Charles University, Prague, Czechia; ^2^Department of Botany, University of British Columbia, Vancouver, BC, Canada; ^3^Department of Parasitology, Faculty of Science, Charles University, BIOCEV, Vestec, Czechia; ^4^Polar-Geo-Lab, Department of Geography, Faculty of Science, Masaryk University, Brno, Czechia; ^5^Regional Museum in Jeseník, Jeseník, Czechia; ^6^Laboratory of Cell Biology, Institute of Experimental Botany of the Czech Academy of Sciences, Prague, Czechia; ^7^Department of Experimental Plant Biology, Faculty of Science, Charles University, Prague, Czechia

**Keywords:** plant evolution, Cryogenian glaciation, Streptophyta, Charophyta, Anydrophyta, Zygnematophyceae, Embryophyta, Snowball Earth

## Abstract

For tens of millions of years (Ma), the terrestrial habitats of Snowball Earth during the Cryogenian period (between 720 and 635 Ma before present–Neoproterozoic Era) were possibly dominated by global snow and ice cover up to the equatorial sublimative desert. The most recent time-calibrated phylogenies calibrated not only on plants but on a comprehensive set of eukaryotes indicate that within the Streptophyta, multicellular charophytes (Phragmoplastophyta) evolved in the Mesoproterozoic to the early Neoproterozoic. At the same time, Cryogenian is the time of the likely origin of the common ancestor of Zygnematophyceae and Embryophyta and later, also of the Zygnematophyceae–Embryophyta split. This common ancestor is proposed to be called Anydrophyta; here, we use anydrophytes. Based on the combination of published phylogenomic studies and estimated diversification time comparisons, we deem it highly likely that anydrophytes evolved in response to Cryogenian cooling. Also, later in the Cryogenian, secondary simplification of multicellular anydrophytes and loss of flagella resulted in Zygnematophyceae diversification as an adaptation to the extended cold glacial environment. We propose that the Marinoan geochemically documented expansion of first terrestrial flora has been represented not only by Chlorophyta but also by Streptophyta, including the anydrophytes, and later by Zygnematophyceae, thriving on glacial surfaces until today. It is possible that multicellular early Embryophyta survived in less abundant (possibly relatively warmer) refugia, relying more on mineral substrates, allowing the retention of flagella-based sexuality. The loss of flagella and sexual reproduction by conjugation evolved in Zygnematophyceae and zygomycetous fungi during the Cryogenian in a remarkably convergent way. Thus, we support the concept that the important basal cellular adaptations to terrestrial environments were exapted in streptophyte algae for terrestrialization and propose that this was stimulated by the adaptation to glacial habitats dominating the Cryogenian Snowball Earth. Including the glacial lifestyle when considering the rise of land plants increases the parsimony of connecting different ecological, phylogenetic, and physiological puzzles of the journey from aquatic algae to terrestrial floras.

## Introduction–The Timing of the Terrestrial Flora Rise

The timing of the evolution of terrestrial flora, the branching of Streptophyta lineages resulting in Embryophyta establishment, and their further diversification have been a subject of interest since the 19th century. The most recent debate is fueled by the emergence of large genomic and transcriptomic datasets (summarized in Rensing, 2020). The categorization of terrestrialization is used differently in different contexts. It is often mixed with the wet-to-dry transition, e.g., in some chlorophyte or streptophyte aerophytic algae (for terrestrialisation in the geology context see further). The wet-to-dry transition of some streptophyte algae might be a very initial part of following terrestrialization. However, during this report, we use the category of terrestrialization *sensu stricto* as only related to the establishment of Embryophyta–land plants–starting, however, in the common ancestor of Embryophyta and Zygnematophyceae–anydrophytes (see further).

The investigation of these ancient events is based on a combination of data sources, and there are apparent challenges linked with their interpretations. Firstly, an almost complete lack of fossil record of transition stages of terrestrialization in addition to the extinction of the close relatives of the early land plants. This was highlighted recently by the support of the monophyly of the Bryophyta as the sister clade to Tracheophyta (Nishiyama et al., 2004; Civáň et al., 2014; Cox et al., 2014; Puttick et al., 2018), leaving a larger gap in our understanding of the land plant evolution. There are still textbooks in use that state the assumption that the Tracheophyta evolved from Bryophyta, which is a scenario now invalidated. Secondly, the uncertainty of the timing estimates (contrasting predictions and large confidence intervals) based on molecular clock reaching deeper back in time. And thirdly, there is difficulty with interpreting an independent source of information on primary production on land provided by the geological/geochemistry record, as it has almost no taxonomic resolution.

### Fossil Evidence of Terrestrialization

Due to the well-acknowledged poor fossilization of plant remnants, spores are considered to be the best early markers of land plant occurrence. Currently, the oldest trilete spores (narrow Y shape lines radiating from a spore surface center indicating an origin in a single meiosis quadruple structure product) assignable to Embryophyta are known from the Upper Ordovician (Steemans et al., 2009; Edwards et al., 2014; Rubinstein and Vajda, 2019), along with poorly preserved seemingly polysporangiate mesofossils (Salamon et al., 2018). The widespread occurrence of non–trilete cryptospores–i.e., variable first spore types of the presumptive terrestrial origin without trilete scar–(Edwards et al., 2014) in the Ordovician, starting in the middle Ordovician (Dapingian), is proposed to be a sign for the great Ordovician diversification of land plants (Servais et al., 2019). Current data support the hypothesis that in the Ordovician, there were already diversified tiny, possibly polysporangiate, cryptophyte-type floras. During the following Silurian, cryptospores producing plants decline while trilete producers prevail (Pšenička et al., 2021). It is important to note that cryptophytes *sensu* Edwards (Edwards et al., 2014) are extinct plants producing cryptospores, not to be confused with Cryptophyceae algae. Therefore the very first adaptations of Streptophyta to the dry land occurred much earlier, most probably in the Precambrian (Hedges et al., 2018; Servais et al., 2019; Shinohara and Nishitani, 2021; Strassert et al., 2021; Su et al., 2021). The first Silurian flora documented by macrofossils (e.g., Cooksonia, Baragwanathia) suggests an undocumented evolution and diversification of land plants preceding the earliest known fossils (Pšenička et al., 2021). We will discuss the potential importance of Cryogenian acritarchs for understanding the evolution of land plants in section “Zygnematophyceae Diverged From Multicellular Anydrophytes Later in the Cryogenian, Adapting to Ice–Dominated Surface” and the “Discussion.”

### Time-Calibrated Phylogeny

**FIGURE 1:**
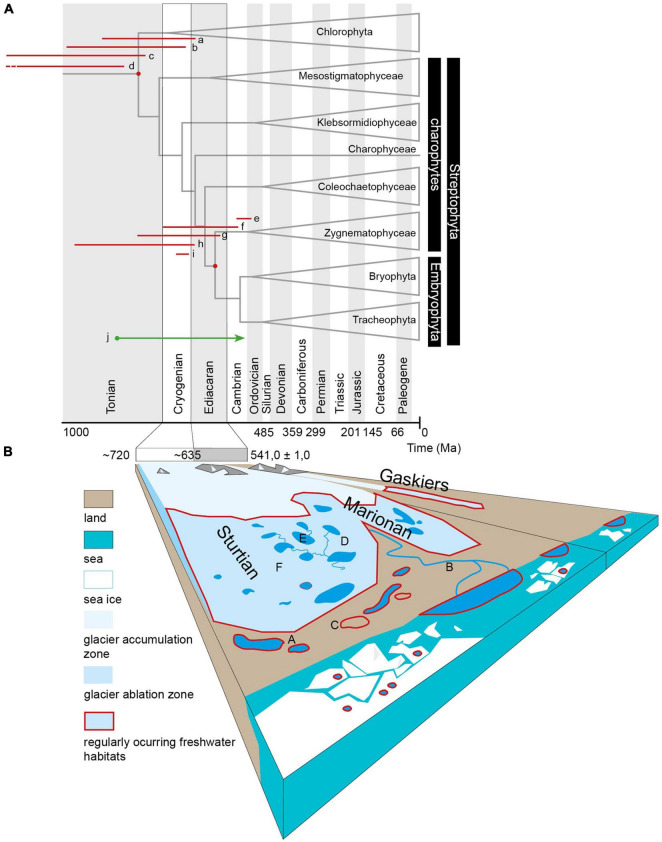
**(A)** The phylogenetic tree with time estimates for the splits of Chlorophyta–Streptophyta and Zygnematophyceae–Embryophyta. Tree adapted from Morris et al. (2018b), projected over timescale based on international geostratigraphic chart (2020). The red lines at the respective node represent 95% highest posterior densities of estimates presented by Hedges et al. (2018), Morris et al. (2018b), Strassert et al. (2021), and Su et al. (2021): a–Streptophyta, Morris et al. (2018b) all calibrations, b–Streptophyta, Hedges et al. (2018) Mesozoic calibrations, c–Streptophyta, Hedges et al. (2018) Spermatophyta calibration, d–Streptophyta, Strassert et al. (2021), e–Embryophyta, Morris et al. (2018b) all calibrations, f–Embryophyta, Hedges et al. (2018) Mesozoic calibrations, g–Embryophyta, Hedges et al. (2018) Spermatophyta calibration, h–Embryophyta, Su et al. (2021), Strassert et al. (2021). j–Influx of terrestrial carbon is apparent in carbonates younger than 850 Ma, according to a study of Knauth and Kennedy (2009), who infer an explosion of photosynthesizing communities on late Precambrian land surfaces. **(B)** Schematic presentation of the potential freshwater habitats in a low latitude Cryogenian catchment. The picture represents habitats populated by members of streptophyte algae in the current biosphere (A) Lentic habitats (*Mesostigma, Chara*), (B) fluvial habitats with various *Zygnematophyceae* in the phytobenthos and e.g., *Coleochaete* on submerged surfaces, (C) subaeric habitats, moist or periodically submerged surfaces, and biological soil crusts (e.g., *Klebsormidium*, *Chlorokybus*, and some *Zygnematophyceae*).

**FIGURE 2:**
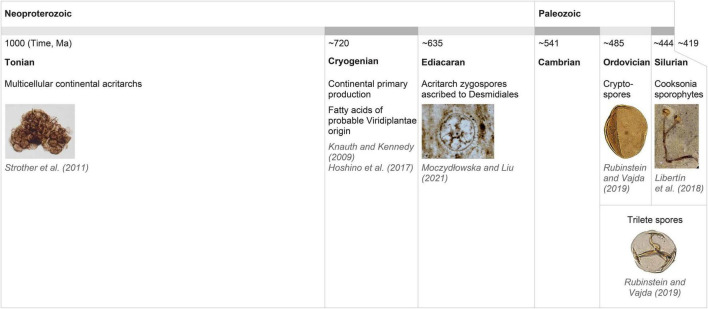
Paleontological and geological evidence for the continental (most probably eukaryotic) organisms and primary production by photosynthetic organisms/plants from the Neoproterozoic through Cryogenian to the early Paleozoic. For our hypothesis, the existence of desmids in the early Ediacaran, as indicated by the Acritarch zygospores ascribed to Desmidiales (Moczydłowska and Liu, 2021), is of central importance.

The land plants (Embryophyta), along with their algal relatives (streptophyte algae), constitute a clade called Streptophyta. Together with the algal group Chlorophyta and a small group Prasinodermatophyta, they constitute the green plants (Viridiplantae; Li et al., 2020). The deepest basal lineage of Streptophyta is formed by the unicellular biflagellate monotypic genera *Mesostigma*, described as a rarely but regularly occurring inhabitant of freshwater phytobenthos in wintertime (Lauterborn, 1894), and an aerophytic colonial monotypic genera *Chlorokybus* (Lemieux et al., 2007). The algal representatives of Streptophyta further include the filamentous Klebsormidiophyceae (Stewart and Mattox, 1975). Klebsormidiophyceae have biflagellate zoospores and combine the features of the early Streptophyta with terrestrial plants due to remarkable stress tolerance of vegetative cells (Holzinger et al., 2014). These three lineages are also referred to as the “lower-branching KCM grade” of Streptophyta (Klebsormidiophyceae, Chlorokybophyceae, and Mesostigmatophyceae, de Vries and Archibald, 2018). A further distinct streptophyte lineage is Charophyceae (Rabenhorst, 1870), characterized by their complex multicellular body architecture, which intuitively led to their traditional placement as the sister group to plants from the 19th century until the early molecular studies (Karol et al., 2001). Together with the lineages Coleochaetophyceae (Delwiche et al., 2002) and Zygnematophyceae (= Conjugatophyceae, Guiry, 2013; Cheng et al., 2019), they are referred to as “higher branching ZCC-grade” of Streptophyta by de Vries and Archibald (2018)–along with Embryophyta called Phragmoplastophyta. The recently generally accepted topology of the streptophyte phylogenetic tree recognizes Zygnematophyceae as the closest living lineage to land plants (Clarke et al., 2011; Wodniok et al., 2011; Wickett et al., 2014; Morris et al., 2018b; Leebens-Mack et al., 2019; Su et al., 2021). Rensing (2020) proposed to call the common ancestor of Zygnematophyceae and Embryophyta “Anydrophyta”–pointing out their key drought-resistant abilities. We use anydrophytes throughout this report instead, as this name is not yet generally accepted as part of the plant systematic nomenclature. New molecular clock analyses (Strassert et al., 2021; Su et al., 2021) date the main splits of the streptophyte lineages and the split between Zygnematophyceae and Embryophyta way earlier than the estimates based on the appearance of the first spores in the fossil material (Figures 1A,2) or even relatively recent influential time-calibrated phylogeny by Morris et al. (2018b). The new time-calibrated phylogenies based on the Viridiplantae (Su et al., 2021) and Eukaryota (Strassert et al., 2021) suggest a likely Cryogenian origin of anydrophytes as well as the split of Zygnematophyceae. This dating was indicated very recently by Shinohara and Nishitani (2021) for Zygnematophyceae and acknowledged as a possibility by Lutzoni et al. (2018) and Jiao et al. (2020).

### Isotopic and Chemical Evidence of Early Land Plants in the Geological Record–Green Cryogenian

There is an independent line of evidence integrating the signal of terrestrial productivity over large temporal and spatial scales, namely, the isotopic composition of marine sediments containing interstitial precipitates with a distinct isotopic fingerprint (Fayek et al., 2001). There is evidence for significant terrestrial primary production from the isotopic analyses of the Neoproterozoic marine interstitial carbonates based on combined oxygen and carbon isotope data (Knauth and Kennedy, 2009; Cole et al., 2020). These studies suggest that the terrestrial expansion of photosynthesizing communities preceded the significant climate perturbations of the Neoproterozoic glaciations. It needs to be noted that the term “terrestrialization” is understood often differently among biologists *versus* geologists (see also a distinction to wet-to-dry transition discussed above). Biologists define it as organisms inhabiting soils, surfaces of rocks–dry land in contrast to water inhabitants, while geologists mean inhabited surface of the whole continents, which also includes aquatic habitats as lakes, rivers, and melting glaciers. The conclusions of Knauth and Kennedy (2009) on the late Precambrian greening are formulated in the latter sense; they compared the signal of exploding terrestrial photosynthesizing communities with phanerozoic terrestrial primary production where there is no doubt about its origin in terrestrial habitats. Moreover, the study of Hoshino et al. (2017) finds that C_29_ 24-ethylsteranes are systematically absent from sediments deposited before the onset of the Snowball Earth events but are present in rocks deposited during and directly after the Marinoan glaciation. These findings imply an origin of stigmasteroid biosynthesis, compounds characteristic of photosynthetic Eukaryota, during the glaciation. The authors concluded that this extended glaciation period was an evolutionary stimulant rather than just a bottleneck (Hoshino et al., 2017). We can thus draw a simplified framework where fossil record shows solid evidence for already complex multicellular land plants (Embryophyta) in the Ordovician/Silurian (Pšenička et al., 2021). Molecular data models extend the possible timing for the origin of the anydrophytes and phylogenetic split between Zygnematophyceae and Embryophyta to either the Mesoproterozoic to early Neoproterozoic (Su et al., 2021), the Cryogenian (Strassert et al., 2021), or the Ediacaran/Cambrian (Morris et al., 2018a; see also Figure 1). Additionally, the rise in terrestrial primary productivity documented in sedimentary geologic isotopic records dates to the Tonian and Cryogenian (Knauth and Kennedy, 2009) or, based on sterol biosynthesis evolution, to the final Marinoan glaciation (Hoshino et al., 2017). It is likely that missing fossil evidence of earlier evolutionary stages of land floras is related not only to their poor ability to fossilize (rather than their absence) but also to the Rodinia continent break up and erosion of the Cryogenian. This allows us to infer that the Cryogenian origin of the first land floras is a highly parsimonious scenario and needs to be further critically considered and developed due to the important implications for the physiology and ecology of early terrestrial floras and extant land plants.

## Paleogeography of the Cryogenian

When searching for critical aspects or features of the environment inhabited by the ancestors of the Embryophyta, speculation is inevitable, except for one generally accepted feature: their freshwater origin (Wodniok et al., 2011; de Vries et al., 2016; for deeper evolution and freshwater see Sánchez-Baracaldo et al., 2017; for review see Rensing, 2020). Thus, we focus on the presence, character, and spatiotemporal extent of the freshwater habitats in the mid to late Neoproterozoic world–especially in the Cryogenian.

The Cryogenian marks the period when the supercontinent Rodinia broke up. At the beginning of the Cryogenian (720 Ma), the first rift basins developed in the continental crust of Rodinia, and in 635 Ma (i.e., at the end of Cryogenian), the continued rifting resulted in the break-up of the supercontinent into several blocks (Li et al., 2013). The early advanced chemical weathering of the Rodinia continent resulted in the sequestration of the atmospheric carbon dioxide (CO_2_) in quantity sufficient for global cooling, resulting in the Snowball Earth glaciation conditions (Hoffman and Schrag, 2002). Under these conditions, the oceans were covered with sea ice, and the glaciers covered most of the Rodinia land surface between 0–70° latitude (Li et al., 2013). There are two major glaciations recognized over the course of Cryogenian; the Sturtian and the Marinoan. The Snowball Earth hypothesis assumes complete glaciation of the Earth’s surface (Hoffman and Schrag, 2002), which would intuitively turn the biotic colonization of the continents impossible. However, as summarized and discussed by Eyles (2008), the Snowball Earth glaciations possibly took place as a series of consecutive geo-tectonically predisposed regional glaciations. In the Marinoan glacial epoch, the sedimentological and geochemical records support a deglaciation interval with the onset of glaciolacustrine, non-glacial lacustrine, and ice-free marine conditions (Wang et al., 2008; Williams et al., 2008; Hoffman et al., 2012, 2017). The continents were possibly not completely glaciated even in the maxima of actual glaciation epochs. Exposed alluvial plains and tidal flats allowed the development of subaeric periglacial zones with permafrost (Retallack et al., 2015; Bai et al., 2020). The constituents of petroleum phytane, pristane (isoprenoid alkanes formed from phytol, a constituent of chlorophyll), and sterane (derived from steroids or sterols) in marine sediments provide evidence that there was no break in the activity of photosynthetic eukaryotes during the Cryogenian glacial epochs (Wang et al., 2008; Hoshino et al., 2017). The Neoproterozoic land system differed from the Phanerozoic by the absence of a continuous vegetation cover with a root system (Bose et al., 2012). The Neoproterozoic land system was thus similar to recent arid regions, or the continental polar areas. The dominant terrestrial environment was alluvial plains with unstable riverbeds even though meandering rivers also occurred (Barkat et al., 2020). Along the coast, there were river deltas and tidal flats where the eolic sedimentation of loess and wadded sands also took place (Williams et al., 2008; Retallack, 2011). Additionally, the surface of melting glaciers must be included within the list of potential freshwater habitats.

Habitats occupied by members of streptophyte algae in the current biosphere are represented in Figure 1. For lentic habitats, *Mesostigma* or *Chara* are typical, lentic to slow fluvial phytobenthos often hosts various *Zygnematophyceae*; and the subaeric, moist, or periodically submerged surfaces are occupied by *Klebsormidium* and also *Chlorokybus* or *Coleochaete* and some *Zygnematophyceae*. To some extent, recent supraglacial habitats represent functional analogs to all three previous types of habitats *via* supraglacial lakes and cryoconite melt ponds as lentic habitats, supraglacial streams as fluvial habitats, and by the melting surface of bare ice as a functional analog to subaeric habitat where desiccation is substituted by freezing as the main physiological stress. The last-named habitat is typically inhabited by zygnematophycean genera *Mesotaenium*, *Ancylonema*, and *Cylindrocystis*, where the first two taxa develop regularly large-scale blooms, e.g., on the Greenland ice sheet, but are reported in the ablation zones of glaciers worldwide (Figure 1B; Williamson et al., 2019).

Any glacial system in or close to balance with a climate, which allows the hydrological cycle to develop, must have an ablation zone where melting periodically occurs on an annual (polar areas) or daily (tropics) basis (Benn and Evans, 2010). For organisms that can cope with the supraglacial conditions, this environment can provide a habitat for long-term survival (Hodson et al., 2008; Anesio and Laybourn-Parry, 2012; Stibal et al., 2012). It includes different kinds of cyanobacteria and algae (Yallop et al., 2012; Lutz et al., 2018; Williamson et al., 2019), but sometimes also moss balls (“glacier mice”) which are able to sustain cold-adapted invertebrates (Zawierucha et al., 2015, 2021) and the populations possibly survive over Holocene timescale (Heusser, 1972). However, in the polar glacial condition, the moss life cycle is disrupted and reduced to a vegetative phase, missing sexual reproduction (e.g., Hotaling et al., 2020) in contrast to glacial Zygnematophyceae (e.g., Permann et al., 2021a,b; Procházková et al., 2021).

## Adaptations to Cold, Snow, Ice, Drought, and High Irradiance Exposed Conditions of Cryogenian and the Evolution of Anydrophytes and Their Cellular Exaptations Facilitating Further Terrestrialization

Plant terrestrialization or land plant evolution started most probably at the single-cell stage of Streptophyta evolution by the first wet-to-dry transition–i.e., before the complex multicellularity evolved (Stebbins and Hill, 1980; Becker, 2013; Harholt et al., 2016). Certain aerophytic Chlorophyta adapted on the cellular level to dry land conditions (i.e., were subject to wet-to-dry transition), most probably even much earlier in the Proterozoic (Strassert et al., 2021; Su et al., 2021; Zhang et al., 2021). This hypothesis was recently further supported by studies of the plant cell wall and stress response evolution (Harholt et al., 2016; de Vries et al., 2018; Jensen et al., 2018). The streptophyte algae were living on the land for some time before the emergence of land plants (Harholt et al., 2016). Similarly, de Vries et al. (2018) show that the embryophyte stress signaling evolved in the algal progenitors of land plants. However, environments similar to our extant freshwater to land transitions (lake shores, periodic water bodies, wet aerophytic algae environments) are considered as possible terrestrialisation biotopes in most published cases (e.g., Fürst-Jansen et al., 2020; Rensing, 2020). Based on our new scenario, Streptophyta dry land adaptation started on a single cell level in the late Mesoproterozoic/Tonian before the Cryogenian glaciations. It continued until the establishment of multicellular lineages of Streptophyta–Charophyceae and Coleochaetophyceae in the Mesoproterozoic or the Tonian (at the beginning of the Neoproterozoic). Here, we argue and hypothesize that the tens of millions of years of prevalently cold or freezing environments of the Cryogenian period is the key environmental factor that crucially shaped future land plant cellular adaptations. These adaptations turned to exaptations in Embryophyta exposed to terrestrial conditions and their transition to the dry land.

Rensing (2020) highlighted the ability to survive desiccation as a key factor in the terrestrial ancestors of Zygnematophyceae and Embryophyta. However, we emphasize that on physical, physiological, and molecular levels, the stress imposed by desiccation has similar consequences on the plant organisms as the stress imposed by freezing. The surfaces of recent glaciers with bare ice inhabited by simple Zygnematophyceae (Williamson et al., 2019) and cryoconite occasionally colonized by long term populations of vegetatively propagating mosses (Heusser, 1972; Belkina and Vilnet, 2015) are a living example of survival and growth strategies on glacial surfaces with possible implications for their common ancestor. Life on a melting glacial surface has one crucial advantage over life in the proglacial area in terms of the predictability of water availability driven by seasonal or daily changes in insolation. The production of liquid water can be facilitated by the attachment of organisms to darker particles with lower albedo than the surrounding ice, as observed in globular mosses (glacier mice) attaching to and accumulating cryoconite sediment (Porter et al., 2008; Belkina and Vilnet, 2015). Alternatively, the same effect can be achieved by lowering the albedo of the cells by production and accumulation of pigments, such as purpurogallin in the case of glacial Zygnematophyceae (Remias et al., 2012). Accepting the supraglacial habitats such as cryoconite pans (Hoffman, 2016), lakes, melting snow, or melting bare ice inhabited by Cryogenian Chlorophyta and Streptophyta as freshwater aquatic habitats (Figure 1B) has important implications for understanding early land plant adaptations and refugia in the Cryogenian context. We emphasize this aspect, especially when generating a hypothetical yet realistic scenario for the survival of multicellular (though small and simple) anydrophytes.

Recent genomic analyses of the basal Streptophyta representatives (*Mesostigma* and *Chlorokybus*) and inference for their common ancestor indicate terrestrial adaptations of the photosynthetic apparatus. This is especially true when considering adaptations to high irradiance (photo-oxidative damage and photorespiration) and transcription factors (TFs) regulating dry land stress responses (Wang et al., 2020). Many studies of both Embryophyta and streptophyte algae indicate a tight coupling between the adaptation to high irradiation and cold and drought stress (e.g., de Vries et al., 2017, 2018; Chen X. et al., 2021). These stresses result in the major energetic cellular disbalance and reactive oxygen species (ROS) production and are best studied in model plants, esp. *Arabidopsis* (e.g., Kilian et al., 2007; Rasmussen et al., 2013). de Vries et al. (2018) systematically compared KCM (basal) v. ZCC (Zygnematophyceae, Coleochaetophyceae, Charophyceae) streptophyte algae and concluded more elaborated stress response, especially toward the cold in ZCC-grade streptophyte algae. The intimate connection between drought and cold/freeze stresses are well documented in C-repeat binding factor/dehydration responsive element binding factor (CBF/DREB – dehydratation-responsive element-binding protein) TFs functional involvement, and it is clear that their evolution was boosted in Streptophyta, especially in the lineage leading to Embryophyta (Jiao et al., 2020; Rensing, 2020). The activation of DREB TFs is positively regulated by the inducer of CBF expression 1 (ICE1) transcriptional regulator, which is a direct target of Open Stomata 1 (OST1) kinase (Zhu, 2016; Chen X. et al., 2021). OST1 (a member of the SNF1-related protein kinases 2/SnRK2 family), which was originally identified for its role in stomatal closure (Mustilli et al., 2002), is the central regulator of the cold signaling pathway *via* the activation of DREB TFs (Lamers et al., 2020; Chen X. et al., 2021). The CKIN2 (A) family of proteins in chlorophyte *Chlamydomonas reinhardtii* is closely related to the land plant SnRK2 and contains a conserved SnRK2 box and abscisic acid (ABA) box (but without relation to ABA-dependent signaling which evolved later in Embryophyta) after the kinase domain (Komatsu et al., 2020). The ABA box of *C. reinhardtii* CKIN2 (A) is smaller and contains fewer acidic residues than in *Arabidopsis*. Obvious SnRK2s with an ABA box similar to land plants have been identified in *Klebsormidium* (Komatsu et al., 2020). The overall conservation of sequence structure and gene expression profile strongly links CKIN2 and abiotic stress response regulation. Indeed, many CKIN2s are expressed in response to abiotic stresses, such as hyperosmolarity, ultraviolet (UV) radiation exposure, and low temperatures (Colina et al., 2019; Komatsu et al., 2020). In response to the cold, SNRK2/OST1 phosphorylates the TFs ICE1, in addition to Basic Transcription Factor3 (BTF3; Ding et al., 2015, 2018). Low-temperature-induced OST1 phosphorylation activity is upregulated independently of the plant hormone ABA, a known inducer of OST1 activity in response to osmotic stress (Ding et al., 2015). The OST1/SNRK2 kinase of land plants evolved early in Streptophyta, and SNRK2 from *Klebsormidium niten*s complements quadruple mutant in moss (Shinozawa et al., 2019). It would therefore be interesting to test its functions during cold adaptation.

Late embryogenesis abundant (LEA) proteins protect other cytoplasmic proteins from aggregation during desiccation, osmotic, and low-temperature stress conditions (Shinde et al., 2012). Among several sub-clades of LEA proteins, LEA2 proteins are not found in chlorophyte algae, suggesting they evolved after the split of Chlorophyta and Streptophyta (Becker et al., 2020). A newly evolved class of LEA proteins might provide essential protection to cellular proteins during cold/freeze stress in addition to desiccation. The anydrophytes evolved two additional subfamilies for better protection (Becker et al., 2020) and we propose that this happened under the environmental pressure of Cryogenian glaciations.

The origin of anydrophytes (and later also the Zygnematophyceae) was linked to several crucial horizontal gene transfers (HGTs)–facilitated by the co-existence of streptophyte algae with different kinds of microbes and fungi on the surfaces of land (Lutzoni et al., 2018; Chen R. et al., 2021; Shinohara and Nishitani, 2021). Three TF families (GRAS, HDKNOX2, and BBR/BPC), a homolog of the PYR/PYL/RCAR-like ABA receptor and genes involved in 1,4 b-xylan formation (GUX1–5, PARVUS) and galactan/RG I pectin synthesis (GALS1-3), were likely gained in the common anydrophyte ancestor of Zygnematophyceae and Embryophyta (Cheng et al., 2019). Interestingly, Jiao et al. (2020) found that the desiccation regulating TFs of the GRAS and DREB families expanded in representatives of Desmidiales, *Penium* (Rensing, 2020).

The evolution of land plant chloroplast–embryoplast–with innovations as resistance to exposure to high light stress and desiccation, transfer of particular genes to the nucleus, and the emergence of additional control mechanisms could be hypothesized as being promoted during the Cryogenian glaciations in anydrophytes and then became exaptations for the ongoing terrestrialization (de Vries et al., 2016). We postulate that an important phase of the evolution of the embryoplast occurred during the Cryogenian and its glaciations. For example, the transfer of specific genes from the plastid to the nuclear genome and the evolution of nuclear-encoded plastid RNA polymerase/NEP (de Vries et al., 2016; Cheng et al., 2019).

The exposure of early streptophyte algae to high irradiance (including UV light) under the prevalent cold glaciation conditions of Cryogenian land surfaces might be potentially linked to the well documented large regulatory overlap in stress gene expression reaction to high irradiance, drought, and cold stresses (de Vries et al., 2018; Chen X. et al., 2021). Moreover, the light signal is necessary to fully develop cold (not heat) acclimation (Catalá et al., 2011). This is best studied in the *Arabidopsis* model, where it was shown that this signaling input into the cold adaptation is mediated by HY5 bZIP TF and the COP1 photomorphogenesis regulator is also possibly involved (Catalá et al., 2011). While COP1 was present already in the Archaeplastida ancestor, the HY5 TF evolved only in the common Chlorophyta and Streptophyta ancestor. Low-temperature pathways stimulated by HY5 include genes encoding chalcone isomerase (CHI), chalcone synthase (CHS), and flavonol synthase (FLS). These are three key enzymes in the anthocyanin biosynthetic pathway, important in cold response and especially in high irradiation protection due to their antioxidative/anti-ROS properties (Saigo et al., 2020). The key enzyme in the phenylpropanoid pathway (PAL) was acquired by horizontal gene transfer (HGT) in Streptophyta (Emiliani et al., 2009; Cheng et al., 2019), and precursors of the lignin biosynthetic pathway were possibly involved in stress responses and biotic interactions in basal Streptophyta (de Vries et al., 2017). Blue–light receptor phototropin (evolved previously in a common ancestor of Viridiplantae – Li et al., 2015) was found to function as a cold sensor in the liverwort *Marchantia polymorpha*, suggesting that low temperature and light cues are integrated immediately at the sensor level (Fujii et al., 2017). Phytochromes, which are crucial for red light signaling and temperature stress signaling, evolved in a common ancestor of Streptophyta (Han et al., 2019; Wang et al., 2020). Data from Angiospermophyta indicate that phytochromes can also contribute to the regulation of the isoprenoid metabolism in response to the temperature shift (Bianchetti et al., 2020).

The detailed experimental analysis summarized above clearly shows that the co-regulation of high light and cold stress responses is present already in basal Streptophyta. This co-regulation led de Vries et al. (2017) to conclude that embryophyte stress signaling had already evolved in streptophyte algae. Data from other studies (de Vries J. et al., 2020; Jiao et al., 2020; Wang et al., 2020) supports the interpretation proposed by de Vries et al. (2017) and Fürst-Jansen et al. (2020). We argue that these basal cellular adaptations to dry land in the streptophyte lineage evolved in a long cold and freezing period of the Cryogenian. Therefore, we postulate that adaptations to cold oligotrophic freshwater and periodic freezing/desiccation high irradiance exposed land conditions were later co-opted as exaptations that allowed multicellular early Embryophyta to continue in the transition to dry land in the warm Ediacaran period (Retallack, 2013).

## Zygnematophyceae Diverged From Multicellular Anydrophytes Later in the Cryogenian, Adapting to Ice–Dominated Surfaces

Current dating (e.g., Jiao et al., 2020; but also above summarized other timing reports) excludes the original hypothesis of Becker (2013), which proposes that the Streptophyta and Chlorophyta split occurred during the Cryogenian, as the Streptophyta lineage emerged most probably before or during the Tonian (e.g., Cheng et al., 2019; Jiao et al., 2020; Strassert et al., 2021). As we propose above–an interesting and appealing possibility opened by these recent timings is that multicellular streptophyte algae evolved at or even before the Tonian, and anydrophytes clade of Streptophyta evolved at the beginning or during the first part of the Cryogenian (Jiao et al., 2020; Strassert et al., 2021). Crucial to the further elaboration of our hypothesis is the dating of the diversification of Zygnematophyceae from anydrophytes to within the Cryogenian (Strassert et al., 2021). We propose that the Cryogenian encompasses, firstly, the anydrophyte evolution from the common multicellular streptophyte algae ancestor (closest relatives are Coleochaetophyceae). Secondly, the diversification into extremely cold-adapted cryophilic early Zygnematophyceae (Conjugatophyceae) and early Embryophyta, later in the Cryogenian. Embryophyta were not reduced to single-cell/filamentous level (see further) and also kept flagellated cells, with sexual processes based on motile gametes. It is possible to speculate that early Embryophyta survived the Cryogenian glaciations as a minority flora in the relatively warmer edaphic refugia. It is reasonable to consider that the Cryogenian continental greening (see above Knauth and Kennedy, 2009; Hoshino et al., 2017) was dominated not only by terrestrial Chlorophyta and single-cell/filamentous streptophytes (Lutzoni et al., 2018) but also, and maybe especially, by anydrophytes and later also extremely well cold-adapted early Zygnematophyceae. Therefore, we propose that the biota after the onset of the Cryogenian should also include streptophyte anydrophytes, which split later during the Cryogenian into extremely cold-adapted and reduced (from multicellular to unicellular status–see following chapter) early Zygnematophyceae and early Embryopyta lineages. This is in contrast to Knauth and Kennedy (2009), who speculate on the expansion of a primitive land biota possibly composed of protists, mosses, fungi, and liverworts starting by 1 Ga ago.

Stebbins and Hill (1980) argued that the same evolutionary constraint of dry land drove the convergent evolution of Zygnematophyceae and zygomycetous fungi (incl. Mucoromycota; former Zygomycota–Naranjo-Ortiz and Gabaldón, 2019b; Chang et al., 2021) *via* the loss of flagella and sexuality by conjugation. “Zygomycetous” refers to a paraphyletic phylum (Zygomycota), and this in turn to a sexual structure, the zygospore, that is common to most lineages ascribed to it (Naranjo-Ortiz and Gabaldón, 2019b). These authors hypothesize that a fungal transition to icy environmental niches acts as a facilitator of the transition from water to the terrestrial environment. They elaborate *via* the following scenario: (i) Snowball Earth created a diversification of niches, including microbial ones. (ii) Fungi moved into glacial environments as zoosporic predators of algae, and (iii) necrotic algal mass supported the evolution of hyphal growth and osmotrophy, loss of flagella, the evolution of conjugation, and resistant zygospores (Naranjo-Ortiz and Gabaldón, 2019b). Here we develop a similar hypothesis, suggesting that the split of conjugating Zygnematophyceae from anydrophytes happened under the same selective pressure of Cryogenian glaciations. This is supported by the evidence that the loss of flagella and sex by conjugation of zygomycetous fungi coincides with the Cryogenian glaciations (Taylor and Berbee, 2006; Lutzoni et al., 2018; Naranjo-Ortiz and Gabaldón, 2019a; Chang et al., 2021). Sexuality based on mere conjugation without flagellated gametes is more effective (and therefore evolutionarily more adaptive) for the life cycle completion in comparison to flagellated gamete mediated sexuality of other Cryogenian land inhabiting organisms. It is generally accepted that in contrast to microbes or single-cell organisms, the lower limit for the completion of the life cycle in multicellular organisms (except for endothermic animals) appears to be between 0 and 2°C (Clarke et al., 2013). Basal bodies and flagella are functionally constrained in glacial conditions. This even applies to snow-adapted chlorophyte flagellates*; Haematococcus pluvialis* become cysts in response to cold stress (de Carpentier et al., 2019), and *Chlamydomonas nivalis* cells resorb their flagella after a temperature downshift (Valledor et al., 2013). The microtubular cytoskeleton in plants is sensitive to cold conditions (Nick, 2013), and it is generally accepted that cold-resistant microtubular networks are much less dynamic, while dynamic microtubules are overly sensitive to the cold (Wallin and Strömberg, 1995).

The overall preference of basal extant Zygnematophyceae to bare ice or extremely oligotrophic low-pH freshwater habitats may be reminiscent of the original early Zygnematophyceae Cryogenian adaptation. This would mean that Chlorophyta (Lutzoni et al., 2018), anydrophytes, early Zygnematophyceae, and early Embryophyta co-evolved and radiated contemporaneously with land fungi already in the Cryogenian. This may also explain an early evolution of symbiosis genes that regulate biotic interactions between plants and microbes in Streptophyta (de Vries and Archibald, 2018; de Vries S. et al., 2020).

Stebbins and Hill (1980), proposed that the conjugation in algae and fungi evolved under the same dry land stress conditions, and independent convergent evolution of conjugation in zygomycetous fungi and Zygnematophyceae supports our conclusions (see above and also the “Discussion”). Moreover, the conjugation among streptophyte algae is a homoplasious feature, resulting from the independent convergent evolution (Cheng et al., 2019), as demonstrated by the streptophyte algal genus *Spirotaenia*, which has been shown to be a member of the Mesostigmatophyceae and not Zygnematophyceae despite conjugation sexual process (Gontcharov and Melkonian, 2004; Wickett et al., 2014)—in both cases potentially linked to glaciation conditions.

Stress resistant zygospores of Zygnematophyceae are covered by surface cell wall sporopollenin-like material (Permann et al., 2021a,b) so we looked into known Cryogenian acritarchs and despite an overall reduction in acritarchs diversity during the Cryogenian (Huntley et al., 2006) few new types of acritarchs appeared, among which it seems to be possible to find forms that can be potentially interpreted as Zygnematophyceae zygospores (Moczydłowska, 2008) even on the ultrastructural level (Moczydłowska et al., 2010). This possibility will undoubtedly require the attention of specialists in the future (see also Discussion–Moczydłowska and Liu, 2021).

## Evolutionary Reduction From Multicellularity of Anydrophytes to Single-Cell or Filamentous Status in Zygnematophyceae

**FIGURE 3:**
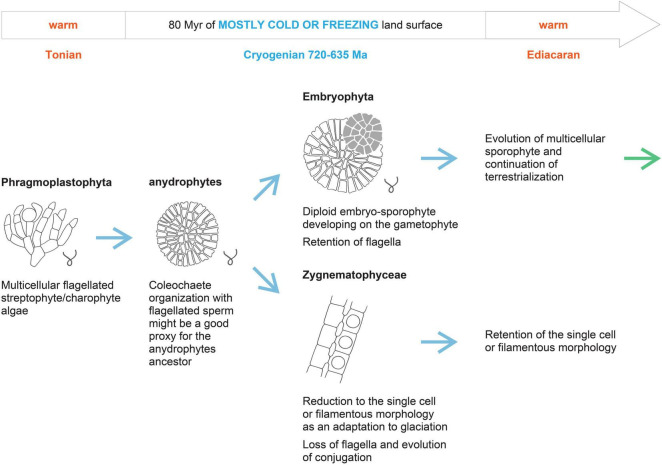
Hypothetical scenario of anydrophyte establishment and Zygnematophyceae split off by the evolutionary reduction under the extended Cryogenian glaciations.

It is generally assumed that Zygnematophyceae evolved by the reduction or loss of morphological complexity, based on the loss of flagella, plasmodesmata, and apical tip growth (Moody, 2020). This assumption is also well supported by the topology of the phylogenetic tree, as both the sister clade of anydrophytes (Coleochaetophyceae) and the sister clade of Zygnematophyceae (Embryophyta) are multicellular. It was proposed that the different mechanisms of cell division between land plants and Zygnematophyceae resulted partially from the simplification of the phragmoplast of the putative common anydrophyte ancestor in the Zygnematophyceae. In more derived Zygnematophyceae, phragmoplast is lost totally (Buschmann and Zachgo, 2016). We used recently published analyses of the early evolution of land plant receptor kinases (Gong and Han, 2021) and several reports on TF evolution to support the idea of secondary Zygnematophyceae simplification. The comparison of independently evolved multicellular organisms indicates evolutionary multiplication of receptor kinases and TFs–both crucial mediators of intercellular communication and tissue differentiation–as a convergent feature of multicellular animals, land plants, Chlorophyta, and brown algae (Cock et al., 2010; de Clerck et al., 2018). Comparing the data from Cheng et al. (2019), Jiao et al. (2020), and Gong and Han (2021), we conclude that along with Lys-M symbiosis-related and S-domain receptor kinases, some others are lost in Zygnematophyceae. Overall, kinase-associated domain types are significantly reduced in Zygnematophyceae compared to *Chara* or Embryophyta (Gong and Han, 2021). However, an exceptionally high number of different types of TFs are retained in single-cell Zygnematophyceae. This might suggest that secondary reductions in structural complexity toward the unicellular status are not necessarily accompanied by reductions in total TF complements as proposed by Jiao et al. (2020). Using the ratio between evolutionary losses and gains of gene orthologous groups as a proxy of evolutionary reduction, it is evident that in Zygnematophyceae, losses prevail–even in contrast to quite a large proportion of losses in *Chara* (Cheng et al., 2019). For *Chara braunii*, there are 834 gains (G) reported against 667 losses (L) (L/G–0.8). While, for *Spirogloea muscicola*, 479 gains are reported against 578 losses (L/G–1.2), and for *Mesotaenium endlicherianum*, 513 gains are reported against 511 losses (L/G–1.0; based on Cheng et al., 2019). These features of Zygnematophyceae compared to sister clades support the concept of secondary reduction of multicellularity of parental anydrophyte clade in the Zygnematophyceae lineage (Figure 3). This secondary simplification of Zygnematophyceae also agrees well with the differences between lower temperature limits for unicellular (−20°C) *versus* multicellular (around 0°C) organisms in respect to the survival and life cycle completion (Clarke et al., 2013) in low temperatures, as discussed above.

## Discussion–Liquid Water to Ice Phase Transition as a Land Plant Evolution Driver

The timing of the beginning of the Cryogenian was shifted from 850 to 720 Ma recently (Shields-Zhou et al., 2015), and the nature of the Cryogenian period is the subject of vivid and ongoing discussions (e.g., Hoffman et al., 2017; Bai et al., 2020). Therefore, data published on the Cryogenian before 2015 (including, e.g., acritarchs) should be carefully considered within the context of the current timing. However, in the context of our hypothesis, the consensus that the Cryogenian was millions of years of extremely cold climate dominated by continental glaciations is sufficient. Scenarios for the climate extremity range from severe “Snowball Earth” to milder–“Slushball Earth” (Hoffman et al., 2017; Bai et al., 2020). With the interruption of more than 10 Ma of warmer interlude, the Cryogenian lasted approximately 80 Ma.

The crucial contentious point for our hypothesis is the timing of major evolutionary splits in the streptophyte lineage. Late dating of Streptophyta establishment to the late Tonian/early Cryogenian and the Zygnematophyceae–Embryophyta split to the late Ediacaran or Cambrian as proposed by Morris et al. (2018b) was challenged directly first by Hedges et al. (2018) and others more recently. Particularly relevant are Jiao et al. (2020), Nie et al. (2020), Moczydłowska and Liu (2021), Strassert et al. (2021) and Su et al. (2021), as these studies used different approaches for molecular clock calibrations or dating. Su et al. (2021) was based solely on Archaeplastida data to compare three dating strategies. Here, it was proposed that the Streptophyta lineage was established in the Paleoproteozoic/Mesoproterozoic, and the anydrophytes emergence and diversification into Zygnematophyceae and Embryophyta occurred in the late Tonian or Cryogenian. Strassert et al. (2021) used a robust approach for their calculations which involved the context of the whole Eukaryota. This resulted in the common ancestors of Coleochaetophyceae and Embryophyta—i.e., the immediate ancestor of anydrophytes–being placed essentially within the Cryogenian era. Su et al. (2021) found that studies favoring a Neoproterozoic origin of land plants (980–682 Ma) are constrained by molecular data while those proposing a Phanerozoic origin (518–500 Ma) are constrained more by the fossil record. They highlighted the important contribution of molecular data (time-dependent molecular change) in the situation of the scarce or very limited fossil record. Importantly, the critical evaluation of how molecular clock-partitioning and fossil calibration strategies affect the uncertainty of evolutionary time scales computation of green plant evolution not only allows but directly indicates possible establishment and diversification of anydrophytes in the Cryogenian (Nie et al., 2020). The time scale ranges of the terrestrial co-evolution of plants and fungi also allow a possibility that the terrestrial colonization by Embryophyta could have occurred as early as 727 Ma (i.e., around the beginning of the Cryogenian; Lutzoni et al., 2018). Timescales by Jiao et al. (2020) also permit the Cryogenian as a possible time of anydrophytes origin and the late Cryogenian as a period of Zygnematophyceae differentiation (Supplementary Table 1E in Jiao et al., 2020). We can therefore conclude that it is reasonable to consider the Cryogenian as a possible time of anydrophyte origin and the diversification of Zygnematophyceae. This is in agreement with the “early” pre-Cryogenian and possibly pre-Tonian timing of the establishment of multicellular streptophyte algae (Su et al., 2021). It also agrees with evidence that Viridiplantae and Rhodophyta were well established by 1,400 Ma (recently based on fossils analyses Zhang et al., 2021; Archaeplastida origin 1,900 Ma, e.g., Strassert et al., 2021), while acritarchs with clear affinity to recent Desmidiales were present in early Ediacaran (Moczydłowska and Liu, 2021; Figure 2 and see also further).

The current plant terrestrialization timing debate (see above, Figure 1), including the emergence of anydrophytes and Zygnematophyceae streptophyte clades, results in consideration of the Cryogenian Snowball Earth, with continents covered by ice, snow, and (periodic) cold freshwater masses as a period relevant to the shaping of plant cellular and developmental adaptations to continental dry land. It is clear that all green algae inhabiting Cryogeninan continents had to adapt to specific chronic glaciations conditions, regardless of the exact timing of Chlorophyta and Streptophyta clades divergences. For example, the case study of *Draparnaldia* chlorophyte aerophytic alga from order Chaetophorales describes how it implements wet-to-dry transition (Caisová, 2020). Recent advancements in deep plant phylogenetics unequivocally resolved *Mesostigma* and *Chlorokybus* as the most basal clade of Streptophyta (Wang et al., 2020). In this context, it is interesting that these extant basal streptophyte algae are oligotrophic, cold-adapted, and are also represented by aerophytic species (*Chlorokybus atmophyticus*). We propose that all early streptophyte lineages living under such conditions were fully adapted to different types of glacial or proglacial environments resulting from tens of Ma of adaptation to the cold Cryogenian. We highlight that they were psychrophilic or cryophilic to different degrees. Current ambient temperature conditions would have been heat stress conditions for them. The evolution of Cryogenian psychrophilic land flora of Chlorophyta, anydrophytes, and later in the Cryogenian also Zygnematophyceae might explain at least part of the geochemical greening of Neoproterozoic (Knauth and Kennedy, 2009; Hoshino et al., 2017), while also contributing to the possible rise in oxygen level before the end of the Cryogenian along with oceanic algal plankton (several models in Cole et al., 2020).

Becker (2013) proposed an interesting hypothesis that Snowball Earth conditions of the long Cryogenian era of the Neoproterozoic were the time of evolutionary split leading to the divergence between the Streptophyta and Chlorophyta lineages. This was based upon the diversification estimates of chlorophyte vs. streptophyte algae in the early 21st century and appreciation of the Cryogenian as an important evolutionary driver for photosynthesis mechanisms. However, we disagree with this scenario because of the improved current time-calibrated phylogeny calculations. Instead, we suggest that the Cryogenian cold and dry continents were the arena for the evolutionary origin of the common Embryophyta–Zygnematophyceae ancestor; anydrophytes. Due to the extended duration of global glaciations, Zygnematophyceae split off from the anydrophytes by simplification as cold-adapted cryophilic flora in later stages of the Cryogenian. Well-established regulatory interconnection between cold/freeze, high light, and drought stress physiological responses is possibly best illustrated by the DREB/CBF TF family regulating drought and cold stress responses (Lamers et al., 2020; Chen X. et al., 2021–see above). Light input into the cold adaptation is mediated by both red light and blue light receptors. It affects the upregulation of anthocyanin antioxidants biosynthesis to protect cells against cold stress enhanced ROS production. This is well documented to be mediated by the DREB/CBF transcriptional regulation in extant land plants (including *Arabidopsis* or *Marchantia*, Fujii et al., 2017; Shi et al., 2018; Bianchetti et al., 2020). This light–cold response coupling feature should also be expected in anydrophytes, as strongly supported by the comparative cold and light stress experiments with basal streptophyte algae *versus* Zygnematophyceae (de Vries et al., 2018).

Inevitably, both green plant lineages–Chlorophyta and Streptophyta–inhabiting continents’ surfaces during the Cryogenian era had to pass through the same extended environmental bottleneck. These constraints resulted in the evolution of important cellular and physiological adaptations (wet-to-dry transitions; see above), which then became exaptations within the streptophyte lineage in the early Embryophyta for the further course of actual terrestrialization in the warmer Ediacaran and early Paleozoic. This agrees with the original hypothesis of Stebbins and Hill (1980), updated by Harholt et al. (2016)–that transition/adaptation to land started already on the single-cell level. It is also experimentally supported by de Vries et al. (2018), showing that terrestrialisation might have started on the single-cell level in algae by wet-to-dry transitions.

However, for our point here, it is important that single cell or filamentous Zygnematophyceae evolved from a multicellular ancestor by a reductive process, so that features relevant for the dry land adaptations described specifically for “ZCC-grade” of Streptophyta (de Vries and Archibald, 2018; see above) are not a result of evolution starting at the single-cell level of streptophyte algal organization. In this respect, it is very interesting that Bowles et al. (2020) recently proposed, based on phylogenomic comparative analyses, the separation of first the evolutionary origins of multicellularity and only later, as the second phase, the terrestrialization in Embryophyta. Our hypothesis that multicellular streptophyte algae evolved into anydrophytes, and were reduced to single-cell/filamentous organization in Zygnematophyceae during the Cryogenian while making crucial dry land adaptations during this epoch agrees with this two-step scenario of Bowles et al. (2020). However, we consider that dry land adaptation started by wet-to-dry transitions in streptophyte algae already on a single-cell level in the late Mesoproterozoic–early Neoproterozoic/Tonian (i.e., before the Cryogenian glaciations) and led to the establishment of freshwater multicellular streptophyte algae in the Mesoproterozoic or Tonian (see timings above and Strother et al., 2011; Figure 2). This means that streptophytes entered the Cryogenian not only as wet-to-dry adapted single cell/colonial or filamentous algae (*Chlorokybus*, *Klebsormidium*) but already also as multicellular organisms (e.g., extant *Chara or Coleochaete*). Anydrophytes, therefore, evolved as an adaptation of already multicellular streptophyte algae to the Cryogenian harsh glacial conditions. Zygnematophyceae evolved by secondary simplification from anydrophytes still in the Cryogenian as cryophilic algae with a life cycle (i.e., sexual process) optimized for cold and dry/freezing conditions (Figure 3). This was due to an extended selective pressure of 80 Ma of the Cryogenian glacial conditions. It is accepted that in the same period, zygomycetous fungi (originally Zygomycota) also lost the flagella and evolved conjugation as a mechanism of sexual reproduction (Liu et al., 2006; Taylor and Berbee, 2006; Lutzoni et al., 2018; Naranjo-Ortiz and Gabaldón, 2019b; Chang et al., 2021). The hypothesis that the same selective pressure of dry land stress conditions resulted in an independent evolution of conjugation in algae and fungi was originally proposed by Stebbins and Hill (1980). We believe that an independent convergent (homoplasy) evolution of conjugation in zygomycetous fungi and Zygnematophyceae supports our whole concept; Zygnematophyceae evolved under the same Cryogenian selective environmental conditions as zygomycetous fungi. This idea is further supported by an independent evolution of conjugation among the Mesostigmatophyceae (Wickett et al., 2014; see above).

A fascinating example of the possible convergent evolution of cell wall dynamics regulating xyloglucan endotransglucosylase/hydrolases (XTHs) and related enzymes between Zygnematophyceae and zygomycetous fungi in Cryogenian was shown recently by Shinohara and Nishitani (2021). Using comparative phylogenomic analyses, they traced the non-plant origins of the XTH family to Alphaproteobacteria, pre-XTH enzymes were integrated into zygnematophycean algae in the Cryogenian *via* the likely HGT. Subsequent HGT event of the zygnematophycean XTHs in the Cryogenian may have led to fungal Congo Red Hypersensitive (CRH) enzymes that cleave and reconnect chitin and glucans in fungal cell walls (name from yeast knockout mutants with increased sensitivity to cell-wall-interfering congo red stain). The HGTs of cell wall plasticity regulating enzyme encoding genes may have supported the adaptation of plants and fungi to the ancient icy environment by facilitating their dry land sessile lifestyles (Shinohara and Nishitani, 2021). In both instances, this might have contributed to the regulation of the conjugation process requiring large local cell wall modifications of conjugating partner gametes.

The emergence of Zygnematophyceae is expected to have contributed to the acritarch diversity (Moczydłowska et al., 2010; see above). Several morphotypes of acritarchs allow zygnematophycean zygospore interpretation. Additionally, during the Tonian and the Cryogenian new types of acritarchs appeared (Agić et al., 2017). A recent report by Moczydłowska and Liu (2021) further supports our hypothesis. This study showed the existence of advanced zygospores ascribed to Desmidiales (i.e., relatively derived Zygnematophyceae) at the beginning of the Ediacaran. Therefore, the timings of anydrophyte emergence and split to Zygnematophyceae *versus* Embryophyta proposed by Morris et al. (2018a,b) are rather improbable also based on the recent fossil record.

We further propose the hypothesis that the reduction of organismal complexity from the characean Phragmoplastophyta and anydrophyte multicellularity to unicellularity or filamentous growth in Zygnematophycea was the result of an original evolutionary adaptation and specialization to glacial conditions. On the other hand, Embryophyta ancestors might have occupied relatively warmer refugia/habitats on Rodinia palaeosols (or cryoconite) around the equatorial latitudes or volcanic areas. The survival and life cycle completion lower temperature limits for unicellular (up to −20°C) *versus* multicellular (around 0°C; Clarke et al., 2013) organisms may have played a crucial role in Zygnematophyceae evolution. In this context, it might not be surprising that the basal aerophytic Zygnematophyceae are cold-tolerant or directly psychro- or cryophilic (e.g., *Mesotaenium* or *Ancylonema*–as examples of typical glacial algae; Williamson et al., 2019; Procházková et al., 2021). The loss of dependence on microtubuli-based motility of gametes during the sexual process could be explained as an adaptation for the persistent cold or freezing conditions and possibly the lack of stable mineral substrate in both zygomycetous fungi as well as Zygnematophyceae (see above).

We propose that anydrophytes emergence in the Cryogenian was a crucial initial event in the terrestrialisation and land plant evolution. The following Zygnematophyceae split off was a result of reductive evolution to glacial habitats based on further cold adaptations during the extended Cryogenian period.

## Data Availability Statement

The original contributions presented in the study are included in the article/supplementary material, further inquiries can be directed to the corresponding author.

## Author Contributions

JŽ and ViŽ coined the initial idea of this hypothesis and were joined from the beginning by VoŽ with expertise in phylogenetic analyses and MH with expertise in polar geology. JŽ, ViŽ, MH, and VoŽ wrote the manuscript. All authors read and approved the submitted manuscript.

## Conflict of Interest

The authors declare that the research was conducted in the absence of any commercial or financial relationships that could be construed as a potential conflict of interest.

## Publisher’s Note

All claims expressed in this article are solely those of the authors and do not necessarily represent those of their affiliated organizations, or those of the publisher, the editors and the reviewers. Any product that may be evaluated in this article, or claim that may be made by its manufacturer, is not guaranteed or endorsed by the publisher.

## References

[B1] Agić H. Moczydłowska M. Yin L. (2017). Diversity of organic-walled microfossils from the early Mesoproterozoic Ruyang Group, North China Craton - A window into the early eukaryote evolution. *Precambrian Res.* 297 101–130. 10.1016/j.precamres.2017.04.042

[B2] Anesio A. N. Laybourn-Parry J. (2012). Glaciers and ice sheets as a biome. *Trends Ecol. Evol.* 27 219–225. 10.1016/j.tree.2011.09.012 22000675

[B3] Bai H. Kuang H. Liu Y. Peng N. Chen X. Yuchong Wang Y. (2020). Marinoan-aged red beds at Shennongjia, South China: evidence against global-scale glaciation during the Cryogenian. *Palaeogeogr. Palaeoclimatol. Palaeoecol.* 559:109967. 10.1016/j.palaeo.2020.109967

[B4] Barkat R. Chakraborty P. P. Saha S. Das K. (2020). Alluvial architecture, paleohydrology and provenance tracking from the Neoproterozoic Banganapalle formation, Kurnool Group, India: an example of continental sedimentation before land plants. *Precambrian Res.* 350:105930. 10.1016/j.precamres.2020.105930

[B5] Becker B. (2013). Snow ball earth and the split of Streptophyta and Chlorophyta. *Trends Plant Sci.* 18 180–183. 10.1016/j.tplants.2012.09.010 23102566

[B6] Becker B. Feng X. Yin Y. Holzinger A. (2020). Desiccation tolerance in streptophyte algae and the algae to land plant transition: evolution of LEA and MIP protein families within the Viridiplantae. *J. Exp. Bot.* 71 3270–3278. 10.1093/jxb/eraa105 32107542 PMC7289719

[B7] Belkina O. A. Vilnet A. A. (2015). Some aspects of the moss population development on the Svalbard glaciers. *Czech. Polar Rep.* 5 160–175. 10.5817/CPR2015-2-14

[B8] Benn D. I. Evans D. J. A. (2010). *Glaciers And Glaciation*, Second Edn. London: Hodder Education.

[B9] Bianchetti R. De Luca B. de Haro L. A. Rosado D. Demarco D. Conte M. (2020). Phytochrome-dependent temperature perception modulates isoprenoid metabolism. *Plant Physiol.* 183 869–882. 10.1104/pp.20.00019 32409479 PMC7333726

[B10] Bose P. K. Eriksson P. G. Sarkar S. Wright D. T. Samanta P. Mukhopadhyay S. (2012). Sedimentation patterns during the Precambrian: a unique record? *Mar. Pet. Geol.* 33 34–68. 10.1016/j.marpetgeo.2010.11.002

[B11] Bowles A. M. C. Bechtold U. Paps J. (2020). The origin of land plants is rooted in two bursts of genomic novelty. *Curr Biol.* 30 530–536.e2. 10.1016/j.cub.2019.11.090 31956023

[B12] Buschmann H. Zachgo S. (2016). The evolution of cell division: from streptophyte algae to land plants. *Trends Plant Sci.* 21 872–883. 10.1016/j.tplants.2016.07.004 27477927

[B13] Caisová L. (2020). *Draparnaldia*: a chlorophyte model for comparative analyses of plant terrestrialisation. *J. Exp. Bot.* 71 3305–3313. 10.1093/jxb/eraa102 32100007

[B14] Catalá R. Medina J. Salinas J. (2011). Integration of low temperature and light signaling during cold acclimation response in *Arabidopsis*. *Proc. Natl. Acad. Sci. U. S. A.* 27 16475–16480. 10.1073/pnas.1107161108 21930922 PMC3182711

[B15] Chang Y. Rochon D. Sekimoto S. Wang Y. Chovatia M. Sandor L. (2021). Genome-scale phylogenetic analyses confirm *Olpidium* as the closest living zoosporic fungus to the non-flagellated, terrestrial fungi. *Sci. Rep.* 11:3217. 10.1038/s41598-021-82607-4 33547391 PMC7865070

[B16] Chen X. Ding Y. Yang Y. Song C. Wang B. Yang S. (2021). Protein kinases in plant responses to drought, salt, and cold stress. *J. Integr. Plant Biol.* 63 53–78. 10.1111/jipb.13061 33399265

[B17] Chen R. Huangfu L. Lu Y. Fang H. Xu Y. Li P. (2021). Adaptive innovation of green plants by horizontal gene transfer. *Biotechnol Adv.* 46:107671. 10.1016/j.biotechadv.2020.107671 33242576

[B18] Cheng S. Xian W. Fu Y. Marin B. Keller J. Wu T. (2019). Genomes of subaerial Zygnematophyceae provide insights into land plant evolution. *Cell* 179 1057–1067.e14. 10.1016/j.cell.2019.10.019 31730849

[B19] Civáň P. Foster P. G. Embley M. T. Seneca A. Cox C. J. (2014). Analyses of Charophyte chloroplast genomes help characterise the ancestral chloroplast genome of land plants. *Genome Biol. Evol.* 6 897–911. 10.1093/gbe/evu061 24682153 PMC4007539

[B20] Clarke A. Morris G. J. Fonseca F. Murray B. J. Acton E. Price H. C. (2013). A low temperature limit for life on Earth. *PLoS One* 8:e66207. 10.1371/journal.pone.0066207 23840425 PMC3686811

[B21] Clarke J. T. Warnock R. C. M. Donoghue P. C. J. (2011). Establishing a time-scale for plant evolution. *New Phytol.* 192 266–301. 10.1111/j.1469-8137.2011.03794.x 21729086

[B22] Cock J. M. Sterck L. Rouzé P. Scornet D. Allen A. E. Amoutzias G. (2010). The *Ectocarpus* genome and the independent evolution of multicellularity in brown algae. *Nature* 465 617–621. 10.1038/nature09016 20520714

[B23] Cole D. B. Mills D. B. Erwin D. H. Sperling E. A. Porter S. M. Reinhard C. T. (2020). On the co-evolution of surface oxygen levels and animals. *Geobiology* 18 260–281. 10.1111/gbi.12382 32175670

[B24] Colina F. Amaral J. Carbó M. Pinto G. Soares A. Cañal M. J. (2019). Genome-wide identification and characterisation of CKIN/SnRK gene family in *Chlamydomonas reinhardtii*. *Sci. Rep.* 9:350. 10.1038/s41598-018-35625-8 30674892 PMC6344539

[B25] Cox C. J. Li B. Foster P. G. Embley T. M. Civáò P. (2014). Conflicting phylogenies for early land plants are caused by composition biases among synonymous substitutions. *Syst. Biol.* 63 272–279. 10.1093/sysbio/syt109 24399481 PMC3926305

[B26] de Carpentier F. Lemaire S. D. Danon A. (2019). When unity is strength: the strategies used by *Chlamydomonas* to survive environmental stresses. *Cells* 11:1307. 10.3390/cells8111307 31652831 PMC6912462

[B27] de Clerck O. Kao S. M. Bogaert K. A. Blomme J. Foflonker F. Kwantes M. (2018). Insights into the evolution of multicellularity from the sea lettuce genome. *Curr. Biol.* 28 2921–2933.e5. 10.1016/j.cub.2018.08.015 30220504

[B28] de Vries J. Archibald J. M. (2018). Plant evolution: landmarks on the path to terrestrial life. *New Phytol.* 217 1428–1434. 10.1111/nph.14975 29318635

[B29] de Vries J. Curtis B. A. Gould S. B. Archibald J. M. (2018). Embryophyte stress signaling evolved in the algal progenitors of land plants. *Proc. Natl. Acad. Sci. U. S. A.* 115 3471–3480. 10.1073/pnas.1719230115 29581286 PMC5899452

[B30] de Vries J. de Vries S. Slamovits C. H. Rose L. E. Archibald J. M. (2017). How embryophytic is the biosynthesis of phenylpropanoids and their derivatives in streptophyte Algae? *Plant Cell Physiol.* 58 934–945. 10.1093/pcp/pcx037 28340089

[B31] de Vries J. Stanton A. Archibald J. M. Gould S. B. (2016). Streptophyte terrestrialisation in light of plastid evolution. *Trends Plant Sci.* 21 467–476. 10.1016/j.tplants.2016.01.021 26895731

[B32] de Vries S. Stukenbrock E. H. Rose L. E. (2020). Rapid evolution in plant-microbe interactions - an evolutionary genomics perspective. *New Phytol.* 226 1256–1262. 10.1111/nph.16458 31997351

[B33] de Vries J. de Vries S. Curtis B. A. Zhou H. Penny S. Feussner K. (2020). Heat stress response in the closest algal relatives of land plants reveals conserved stress signaling circuits. *Plant J.* 103 1025–1048. 10.1111/tpj.14782 32333477

[B34] Delwiche C. F. Karol K. G. Cimino M. T. Sytsma K. J. (2002). Phylogeny of the genus *Coleochaete* (Coleochaetales, Charophyta) and related taxa inferred by analysis of the chloroplast gene rbcL. *J. Phycol.* 38 394–403. 10.1046/j.1529-8817.2002.01174.x

[B35] Ding Y. Jia Y. Shi Y. Zhang X. Song C. Gong Z. (2018). OST1-mediated BTF3L phosphorylation positively regulates CBFs during plant cold responses. *EMBO J.* 37:e98228. 10.15252/embj.201798228 29507081 PMC5897776

[B36] Ding Y. Li H. Zhang X. Xie Q. Gong Z. Yang S. (2015). OST1 kinase modulates freezing tolerance by enhancing ICE1 stability in *Arabidopsis*. *Dev. Cell* 32 278–289. 10.1016/j.devcel.2014.12.023 25669882

[B37] Edwards D. Morris J. L. Richardson J. B. Kenrick P. (2014). Cryptospores and cryptophytes reveal hidden diversity in early land floras. *New Phytol.* 202 50–78. 10.1111/nph.12645 24410730

[B38] Emiliani G. Fondi M. Fani R. Gribaldo S. (2009). A horizontal gene transfer at the origin of phenylpropanoid metabolism: a key adaptation of plants to land. *Biol. Direct.* 4:7. 10.1186/1745-6150-4-7 19220881 PMC2657906

[B39] Eyles N. (2008). Glacio-epochs and the supercontinent cycle after ∼3.0 Ga: tectonic boundary conditions for glaciation. *Palaeogeogr. Palaeoclimatol. Palaeoecol.* 258 89–129. 10.1016/j.palaeo.2007.09.021

[B40] Fayek M. Harrison T. M. Grove M. Mckeegan K. D. Coath C. D. Boles J. R. (2001). *In situ* stable isotopic evidence for protracted and complex carbonate cementation in a petroleum reservoir, North Coles Levee, San Joaquin basin, California, U.S.A. *J. Sediment. Res.* 71 444–458. 10.1306/2DC40954-0E47-11D7-8643000102C1865D

[B41] Fujii Y. Tanaka H. Konno N. Ogasawara Y. Hamashima N. Tamura S. (2017). Phototropin perceives temperature based on the lifetime of its photoactivated state. *Proc. Natl. Acad. Sci. U. S. A.* 114 9206–9211. 10.1073/pnas.1704462114 28784810 PMC5576800

[B42] Fürst-Jansen J. M. R. de Vries S. de Vries J. (2020). Evo-physio: on stress responses and the earliest land plants. *J. Exp. Bot.* 71 3254–3269. 10.1093/jxb/eraa007 31922568 PMC7289718

[B43] Gong Z. Han G. Z. (2021). Flourishing in water: the early evolution and diversification of plant receptor-like kinases. *Plant J.* 106 174–184. 10.1111/tpj.15157 33423360

[B44] Gontcharov A. A. Melkonian M. (2004). Unusual position of the genus *Spirotaenia* (Zygnematophyceae) among streptophytes revealed by SSU rDNA and rbcL sequence comparisons. *Phycologia* 43 105–113. 10.2216/i0031-8884-43-1-105.1

[B45] Guiry M. D. (2013). Taxonomy and nomenclature of the Conjugatophyceae (= Zygnematophyceae). *Algae* 28 1–29. 10.4490/algae.2013.28.1.001

[B46] Han X. Chang X. Zhang Z. Chen H. He H. Zhong B. (2019). Origin and evolution of core components responsible for monitoring light environment changes during plant terrestrialization. *Mol. Plant* 12 847–862. 10.1016/j.molp.2019.04.006 31009752

[B47] Harholt J. Moestrup Ø Ulvskov P. (2016). Why plants were terrestrial from the beginning. *Trends Plant Sci.* 21 96–101. 10.1016/j.tplants.2015.11.010 26706443

[B48] Hedges S. B. Tao Q. Walker M. Kumar S. (2018). Accurate timetrees require accurate calibrations. *Proc. Natl. Acad. Sci. U. S. A.* 115 9510–9511. 10.1073/pnas.1812558115 30266795 PMC6187123

[B49] Heusser C. J. (1972). Polsters of the moss *Drepanocladus berggrenii* on gilkey glacier, alaska. *Bull. Torrey Bot. Club* 99 34–36. 10.2307/2484240

[B50] Hodson A. Anesio A. M. Tranter M. Fountain A. Osborn M. Priscu J. (2008). Glacial ecosystems. *Ecol. Monogr.* 78 41–67. 10.1890/07-0187.1

[B51] Hoffman P. F. (2016). Cryoconite pans on Snowball Earth: supraglacial oases for Cryogenian eukaryotes? *Geobiology* 14 531–542. 10.1111/gbi.12191 27422766

[B52] Hoffman P. F. Abbot D. S. Ashkenazy Y. Benn D. I. Brocks J. J. Cohen P. A. (2017). Snowball Earth climate dynamics and Cryogenian geology-geobiology. *Sci. Adv.* 3:e1600983. 10.1126/sciadv.1600983 29134193 PMC5677351

[B53] Hoffman P. F. Halverson G. P. Domack E. W. Maloof A. C. Swanson-Hysell N. L. Cox G. M. (2012). Cryogenian glaciations on the southern tropical paleomargin of Laurentia (NE Svalbard and East Greenland), and a primary origin for the upper Russøya (Islay) carbon isotope excursion. *Precambrian Res.* 206-207 137–158. 10.1016/j.precamres.2012.02.018

[B54] Hoffman P. F. Schrag D. P. (2002). The snowball Earth hypothesis: testing the limits of global change. *Terra Nova.* 14 129–155. 10.1046/j.1365-3121.2002.00408.x

[B55] Holzinger A. Kaplan F. Blaas K. Zechmann B. Komsic-Buchmann K. Becker B. (2014). Transcriptomics of desiccation tolerance in the Streptophyte green alga *Klebsormidium* reveal a land plant-like defense reaction. *PLoS One* 9:e110630. 10.1371/journal.pone.0110630 25340847 PMC4207709

[B56] Hoshino Y. Poshibaeva A. Meredith W. Snape C. Poshibaev V. Versteegh G. J. M. (2017). Cryogenian evolution of stigmasteroid biosynthesis. *Sci. Adv.* 3:e1700887. 10.1126/sciadv.1700887 28948220 PMC5606710

[B57] Hotaling S. Bartholomaus T. C. Gilbert S. L. (2020). Rolling stones gather moss: movement and longevity of moss balls on an Alaskan glacier. *Polar Biol.* 43 735–744. 10.1007/s00300-020-02675-6

[B58] Huntley J. W. Xiao S. Kowalewski M. (2006). “On the morphological history of proterozoic and cambrian acritarchs,” in *Neoproterozoic Geobiology and Paleobiology. Topics in Geobiology*, eds Xiao S. Kaufman A. J. (New York: Springer), 23–56. 10.1007/1-4020-5202-2_2

[B59] Jensen J. K. Busse-Wicher M. Poulsen C. P. Fangel J. U. Smith P. J. Yang J. Y. (2018). Identification of an algal xylan synthase indicates that there is functional orthology between algal and plant cell wall biosynthesis. *New Phytol.* 218 1049–1060. 10.1111/nph.15050 29460505 PMC5902652

[B60] Jiao C. Sørensen I. Sun X. Sun H. Behar H. Alseekh S. (2020). The *Penium margaritaceum* genome: hallmarks of the origins of land plants. *Cell* 181 1097–1111.e12. 10.1016/j.cell.2020.04.019 32442406

[B61] Karol K. G. McCourt R. M. Cimino M. T. Delwiche C. F. (2001). The closest living relatives of land plants. *Science* 294 2351–2353. 10.1126/science.1065156 11743201

[B62] Kilian J. Whitehead D. Horak J. Wanke D. Weinl S. Batistic O. (2007). The AtGenExpress global stress expression data set: protocols, evaluation and model data analysis of UV-B light, drought and cold stress responses. *Plant J.* 50 347–363. 10.1111/j.1365-313X.2007.03052.x 17376166

[B63] Knauth L. P. Kennedy M. J. (2009). The late precambrian greening of the earth. *Nature* 460 728–732. 10.1038/nature08213 19587681

[B64] Komatsu K. Takezawa D. Sakata Y. (2020). Decoding ABA and osmostress signalling in plants from an evolutionary point of view. *Plant Cell Environ.* 43 2894–2911. 10.1111/pce.13869 33459424

[B65] Lamers J. van der Meer T. Testerink C. (2020). How plants sense and respond to stressful environments. *Plant Physiol.* 182 1624–1635. 10.1104/pp.19.01464 32132112 PMC7140927

[B66] Lauterborn R. (1894). Ueber die winterfauna einiger gewässer der oberrheinebene. mit beschreibungen neuer protozoën. *Biol. Zentralblatt* 14 390–398.

[B67] Leebens-Mack J. H. Barker M. S. Carpenter E. J. Deyholos M. K. Gitzendanner M. A. Graham S. W. (2019). One thousand plant transcriptomes and the phylogenomics of green plants. *Nature* 574 679–685. 10.1038/s41586-019-1693-2 31645766 PMC6872490

[B68] Lemieux C. Otis C. Turmel M. (2007). A clade uniting the green algae *Mesostigma viride* and *Chlorokybus atmophyticus* represents the deepest branch of the Streptophyta in chloroplast genome-based phylogenies. *BMC Biol.* 5:2. 10.1186/1741-7007-5-2 17222354 PMC1781420

[B69] Li F. W. Rothfels C. J. Melkonian M. Villarreal J. C. Stevenson D. W. Graham S. W. (2015). The origin and evolution of phototropins. *Front. Plant Sci.* 12:637. 10.3389/fpls.2015.00637 26322073 PMC4532919

[B70] Li L. Z. Wang S. Sahu S. K. Marin B. Li H. Y. Xu Y. (2020). The genome of *Prasinoderma coloniale* unveils the existence of a third phylum within green plants. *Nat. Ecol. Evol.* 4 1220–1231. 10.1038/s41559-020-1221-7 32572216 PMC7455551

[B71] Li Z.-X. Evans D. A. Halverson G. P. (2013). Neoproterozoic glaciations in a revised global palaeogeography from the breakup of Rodinia to the assembly of Gondwanaland. *Sediment. Geol.* 294 219–232. 10.1016/j.sedgeo.2013.05.016

[B72] Liu Y. J. Hodson M. C. Hall B. D. (2006). Loss of the flagellum happened only once in the fungal lineage: phylogenetic structure of Kingdom Fungi inferred from RNA polymerase II subunit genes. *BMC Evol. Biol.* 6:74. 10.1186/1471-2148-6-74 17010206 PMC1599754

[B73] Lutz S. McCutcheon J. McQuaid J. B. Benning L. G. (2018). The diversity of ice algal communities on the Greenland Ice Sheet as revealed by oligotyping. *Microb. Genomics.* 4:e000159. 10.1099/mgen.0.000159 29547098 PMC5885011

[B74] Lutzoni F. Nowak M. D. Alfaro M. E. Reeb V. Miadlikowska J. Krug M. (2018). Contemporaneous radiations of fungi and plants linked to symbiosis. *Nat. Commun.* 9:5451. 10.1038/s41467-018-07849-9 30575731 PMC6303338

[B75] Moczydłowska M. (2008). The Ediacaran microbiota and the survival of Snowball Earth conditions. *Precambrian Res.* 167 1–15. 10.1016/j.precamres.2008.06.008

[B76] Moczydłowska M. Liu P. (2021). Ediacaran algal cysts from the Doushantuo formation, South China. *Geol. Mag.* 158, 1–21. 10.1017/S0016756820001405

[B77] Moczydłowska M. Schopf J. W. Willman S. (2010). Micro- and nano-scale ultrastructure of cell walls in Cryogenian microfossils: revealing their biological affinity. *Lethaia* 43 129–136. 10.1111/j.1502-3931.2009.00175.x

[B78] Moody L. A. (2020). Three-dimensional growth: a developmental innovation that facilitated plant terrestrialisation. *J. Plant Res.* 133 283–290. 10.1007/s10265-020-01173-4 32095969 PMC7214384

[B79] Morris J. L. Puttick M. N. Clark J. W. Edwards D. Kenrick P. Pressel S. (2018b). The timescale of early land plant evolution. *Proc. Natl. Acad. Sci. U. S. A.* 115 E2274–E2283. 10.1073/pnas.1719588115 29463716 PMC5877938

[B80] Morris J. L. Puttick M. N. Clark J. W. Edwards D. Kenrick P. Pressel S. (2018a). Reply to Hedges et al.: accurate timetrees do indeed require accurate calibrations. *Proc. Natl. Acad. Sci. U. S. A.* 115 E9512–E9513. 10.1073/pnas.1812816115 30266794 PMC6187173

[B81] Mustilli A. C. Merlot S. Vavasseur A. Fenzi F. Giraudat J. (2002). *Arabidopsis* OST1 protein kinase mediates the regulation of stomatal aperture by abscisic acid and acts upstream of reactive oxygen species production. *Plant Cell* 14 3089–3099. 10.1105/tpc.007906 12468729 PMC151204

[B82] Naranjo-Ortiz M. A. Gabaldón T. (2019b). Fungal evolution: major ecological adaptations and evolutionary transitions. *Biol. Rev. Camb. Philos. Soc.* 94 1443–1476. 10.1111/brv.12510 31021528 PMC6850671

[B83] Naranjo-Ortiz M. A. Gabaldón T. (2019a). Fungal evolution: diversity, taxonomy and phylogeny of the Fungi. *Biol. Rev.* 94 2101–2137. 10.1111/brv.12550 31659870 PMC6899921

[B84] Nick P. (2013). Microtubules, signalling and abiotic stress. *Plant J.* 75 309–323. 10.1111/tpj.12102 23311499

[B85] Nie Y. Foster C. S. P. Zhu T. Yao R. Duchêne D. A. Ho S. Y. W. (2020). Accounting for uncertainty in the evolutionary timescale of green plants through clock-partitioning and fossil calibration strategies. *Syst. Biol.* 69 1–16. 10.1093/sysbio/syz032 31058981

[B86] Nishiyama T. Wolf P. G. Kugita M. Sinclair R. B. Sugita M. Sugiura C. (2004). Chloroplast phylogeny indicates that bryophytes are monophyletic. *Mol. Biol. Evol.* 21 1813–1819. 10.1093/molbev/msh203 15240838

[B87] Permann C. Herburger K. Felhofer M. Gierlinger N. Lewis L. A. Holzinger A. (2021a). Induction of conjugation and cygospore cell wall characteristics in the Alpine *Spirogyra mirabilis* (Zygnematophyceae, Charophyta): advantage under climate change scenarios? *Plants* 10:1740. 10.3390/plants10081740 34451785 PMC8402014

[B88] Permann C. Herburger K. Niedermeier M. Felhofer M. Gierlinger N. Holzinger A. (2021b). Cell wall characteristics during sexual reproduction of *Mougeotia* sp. (Zygnematophyceae) revealed by electron microscopy, glycan microarrays and RAMAN spectroscopy. *Protoplasma* 258 1261–1275. 10.1007/s00709-021-01659-5 33974144 PMC8523461

[B89] Porter P. R. Evans A. J. Hodson A. J. Lowe A. T. Crabtree M. D. (2008). Sediment-moss interactions on a temperate glacier: falljökull, Iceland. *Ann. Glaciol.* 48 25–31. 10.3189/172756408784700734

[B90] Procházková L. Řezanka T. Nedbalová L. Remias D. (2021). Unicellular versus filamentous: the glacial alga *Ancylonema alaskana* comb. et stat. nov. and its ecophysiological relatedness to *Ancylonema nordenskioeldii* (Zygnematophyceae, Streptophyta). *Microorganisms* 9:1103. 10.3390/microorganisms9051103 34065466 PMC8161032

[B91] Pšenička J. Bek J. Frýda J. Žárský V. Uhlířová M. Štorch P. (2021). Dynamics of Silurian plants as response to climate changes. *Life (Basel)* 11:906. 10.3390/life11090906 34575055 PMC8470493

[B92] Puttick M. N. Morris J. L. Williams T. A. Cox C. J. Edwards D. Kenrick P. (2018). The interrelationships of land plants and the nature of the ancestral Embryophyte. *Curr. Biol.* 28 210–213. 10.1016/j.cub.2018.01.063 29456145

[B93] Rabenhorst G. L. (1870). *Kryptogamen-Flora von Sachsen, der Ober-Lausitz, Thüringen und Nordböhmen, mit Berücksichtigung der benachbarten Länder.* Leipzig: E. Kummer, 10.24355/dbbs.084-200909111218-0

[B94] Rasmussen S. Barah P. Suarez-Rodriguez M. C. Bressendorff S. Friis P. Costantino P. (2013). Transcriptome responses to combinations of stresses in *Arabidopsis*. *Plant Physiol.* 161 1783–1794. 10.1104/pp.112.210773 23447525 PMC3613455

[B95] Remias D. Schwaiger S. Aigner S. Leya T. Stuppner H. Lütz C. (2012). Characterisation of an UV- and VIS-absorbing, purpurogallin-derived secondary pigment new to algae and highly abundant in *Mesotaenium berggrenii* (Zygnematophyceae, Chlorophyta), an extremophyte living on glaciers. *FEMS Microbiol. Ecol.* 79 638–648. 10.1111/j.1574-6941.2011.01245.x 22092588

[B96] Rensing S. A. (2020). How plants conquered land. *Cell* 181 964–966. 10.1016/j.cell.2020.05.011 32470404

[B97] Retallack G. J. (2011). Neoproterozoic loess and limits to snowball Earth. *J. Geol. Soc.* 168 289–308. 10.1144/0016-76492010-051

[B98] Retallack G. J. (2013). Ediacaran life on land. *Nature* 493 89–92. 10.1038/nature11777 23235827

[B99] Retallack G. J. Gose B. N. Osterhout J. T. (2015). Periglacial paleosols and Cryogenian paleoclimate near Adelaide, South Australia. *Precambrian Res.* 263 1–18. 10.1016/j.precamres.2015.03.002

[B100] Rubinstein C. V. Vajda V. (2019). Baltica cradle of early land plants? Oldest record of trilete spores and diverse cryptospore assemblages; evidence from Ordovician successions of Sweden. *GFF* 141 181–190. 10.1080/11035897.2019.1636860

[B101] Saigo T. Wang T. Watanabe M. Tohge T. (2020). Diversity of anthocyanin and proanthocyanin biosynthesis in land plants. *Curr. Opin. Plant Biol.* 55 93–99. 10.1016/j.pbi.2020.04.001 32387820

[B102] Salamon M. A. Gerrienne P. Steemans P. Gorzelak P. Filipiak P. Le Hérissé A. (2018). Putative late ordovician land plants. *New Phytol.* 218 1305–1309. 10.1111/nph.15091 29542135

[B103] Sánchez-Baracaldo P. Raven J. A. Pisani D. Knoll A. H. (2017). Early photosynthetic eukaryotes inhabited low-salinity habitats. *Proc. Natl. Acad. Sci. U. S. A.* 114 E7737–E7745. 10.1073/pnas.1620089114 28808007 PMC5603991

[B104] Servais T. Cascales-Miñana B. Cleal C. J. Gerrienne P. Harper D. A. T. Neumann M. (2019). Revisiting the great Ordovician diversification of land plants: recent data and perspectives. *Palaeogeogr. Palaeoclimatol. Palaeoecol.* 534:109280. 10.1016/j.palaeo.2019.109280

[B105] Shi Y. Ding Y. Yang S. (2018). Molecular regulation of CBF signaling in cold acclimation. *Trends Plant Sci.* 23 623–637. 10.1016/j.tplants.2018.04.002 29735429

[B106] Shields-Zhou G. A. Porter S. Halverson G. P. (2015). A new rock-based definition for the Cryogenian Period (circa 720 - 635 Ma). *Episodes* 39 3–8. 10.18814/epiiugs/2016/v39i1/89231

[B107] Shinde S. Nurul Islam M. Ng C. K. (2012). Dehydration stress-induced oscillations in LEA protein transcripts involves abscisic acid in the moss, *Physcomitrella patens*. *New Phytol.* 195 321–328. 10.1111/j.1469-8137.2012.04193.x 22591374

[B108] Shinohara N. Nishitani K. (2021). Cryogenian origin and subsequent diversification of the plant cell-wall enzyme XTH family. *Plant Cell Physiol.* 10.1093/pcp/pcab093 [Epub Online ahead of print]. 34197607 PMC8711696

[B109] Shinozawa A. Otake R. Takezawa D. Umezawa T. Komatsu K. Tanaka K. (2019). SnRK2 protein kinases represent an ancient system in plants for adaptation to a terrestrial environment. *Commun. Biol.* 2:30. 10.1038/s42003-019-0281-1 30675528 PMC6340887

[B110] Stebbins G. L. Hill G. J. C. (1980). Did multicellular plants invade the land? *Am. Nat.* 115 342–353. 10.1086/283565

[B111] Steemans P. Hérissé A. L. Melvin J. Miller M. A. Paris F. Verniers J. (2009). Origin and radiation of the earliest vascular land plants. *Science* 324 353–353. 10.1126/science.1169659 19372423

[B112] Stewart K. D. Mattox K. R. (1975). Comparative cytology, evolution and classification of the green algae with some consideration of the origin of other organisms with chlorophylls A and B. *Bot. Rev.* 41 104–135. 10.1007/BF02860837

[B113] Stibal M. Šabacká M. Žárský J. (2012). Biological processes on glacier and ice sheet surfaces. *Nat. Geosci.* 5 771–774. 10.1038/ngeo1611

[B114] Strassert J. F. H. Irisarri I. Williams T. A. Burki F. (2021). A molecular timescale for eukaryote evolution with implications for the origin of red algal-derived plastids. *Nat Commun.* 12:1879. 10.1038/s41467-021-22044-z 33767194 PMC7994803

[B115] Strother P. K. Battison L. Brasier M. D. Wellman C. H. (2011). Earth’s earliest non-marine eukaryotes. *Nature* 473 505–509. 10.1038/nature09943 21490597

[B116] Su D. Yang L. Shi X. Ma X. Zhou X. Hedges B. S. (2021). Large-scale phylogenomic analyses reveal the monophyly of bryophytes and Neoproterozoic origin of land plants. *Mol. Biol. Evol.* 38 3332–3344. 10.1093/molbev/msab106 33871608 PMC8321542

[B117] Taylor J. W. Berbee M. L. (2006). Dating divergences in the fungal tree of life: review and new analyses. *Mycologia* 98 838–849. 10.1080/15572536.2006.11832614 17486961

[B118] Valledor L. Furuhashi T. Hanak A. M. Weckwerth W. (2013). Systemic cold stress adaptation of *Chlamydomonas reinhardtii*. *Mol. Cell Proteomics* 12 2032–2047. 10.1074/mcp.M112.026765 23564937 PMC3734567

[B119] Wallin M. Strömberg E. (1995). Cold-stable and cold-adapted microtubules. *Int. Rev. Cytol.* 157 1–31. 10.1016/S0074-7696(08)62155-5 7706018

[B120] Wang S. Li L. Li H. Sahu S. K. Wang H. Xu Y. (2020). Genomes of early-diverging streptophyte algae shed light on plant terrestrialisation. *Nat. Plants* 6 95–106. 10.1038/s41477-019-0560-3 31844283 PMC7027972

[B121] Wang T.-G. Li M. Wang C. Wang G. Zhang W. Shi Q. (2008). Organic molecular evidence in the late neoproterozoic tillites for a palaeo-oceanic environment during the snowball earth era in the yangtze region, southern China. *Precambrian Res.* 162 317–326. 10.1016/j.precamres.2007.09.009

[B122] Wickett N. J. Mirarab S. Nguyen N. Warnow T. Carpenter E. Matasci N. (2014). Phylotranscriptomic analysis of the origin and early diversification of land plants. *Proc. Natl. Acad. Sci. U. S. A.* 111 E4859–E4868. 10.1073/pnas.1323926111 25355905 PMC4234587

[B123] Williams G. E. Gostin V. A. McKirdy D. M. Preiss W. V. (2008). The Elatina glaciation, late Cryogenian (Marinoan Epoch), South Australia: sedimentary facies and palaeoenvironments. *Precambrian Res.* 163 307–331. 10.1016/j.precamres.2007.12.001

[B124] Williamson C. J. Cameron K. A. Cook J. M. Zarsky J. D. Stibal M. Edwards A. (2019). Glacier algae: a dark past and a darker future. *Front. Microbiol.* 10:524. 10.3389/fmicb.2019.00524 31019491 PMC6458304

[B125] Wodniok S. Brinkmann H. Glöckner G. Heidel A. J. Philippe H. Melkonian M. (2011). Origin of land plants: do conjugating green algae hold the key? *BMC Evol. Biol.* 11:104. 10.1186/1471-2148-11-104 21501468 PMC3088898

[B126] Yallop M. L. Anesio A. M. Perkins R. G. Cook J. Telling J. Fagan D. (2012). Photophysiology and albedo-changing potential of the ice algal community on the surface of the Greenland ice sheet. *ISME J.* 6 2302–2313. 10.1038/ismej.2012.107 23018772 PMC3504962

[B127] Zawierucha K. Kolicka M. Takeuchi N. Kaczmarek ł (2015). What animals can live in cryoconite holes? A faunal review: cryoconite holes fauna. *J. Zool.* 295 159–169. 10.1111/jzo.12195

[B128] Zawierucha K. Porazinska D. L. Ficetola G. F. Ambrosini R. Baccolo G. Buda J. (2021). A hole in the nematosphere: tardigrades and rotifers dominate the cryoconite hole environment, whereas nematodes are missing. *J. Zool.* 313 18–36. 10.1111/jzo.12832

[B129] Zhang S. Su J. Ma S. Wang H. Wang X. He K. (2021). Eukaryotic red and green algae populated the tropical ocean 1400 million years ago. *Precambrian Res.* 357:106166. 10.1016/j.precamres.2021.106166

[B130] Zhu J. K. (2016). Abiotic stress signaling and responses in plants. *Cell* 167 313–324. 10.1016/j.cell.2016.08.029 27716505 PMC5104190

